# Editorial: Multimodality imaging of left ventricular assist devices: applications in advanced heart failure

**DOI:** 10.3389/fcvm.2023.1277563

**Published:** 2023-09-28

**Authors:** Valeria Pergola, Matteo Cameli, Michael Dandel, Hatem Soliman-Aboumarie

**Affiliations:** ^1^ Department of Cardiac, Thoracic and Vascular Sciences and Public Health, University of Padua, Padua, Italy; ^2^ Department of Cardiovascular Diseases, University of Siena, Policlinico “Le Scotte”, Siena, Italy; ^3^ Humboldt University Berlin, German Centre for Cardiovascular Research (DZHK), Berlin, Germany; ^4^ Department of Cardiothoracic Intensive Care and Mechanical Circulatory Support, Harefield Hospital, Royal Brompton and Harefield Hospitals, London, United Kingdom; ^5^ School of Cardiovascular and Metabolic Sciences and Medicine, King’s College London, London, United Kingdom

**Keywords:** echocardiagraphy, multimodality imaging, heart failure, cardiac magnet resonance imaging (CMR), cardiac computed tomographic (CT) imaging

**Editorial on the Research Topic** Multimodality imaging of left ventricular assist devices: applications in advanced heart failure

## Introduction

In the realm of medical advancements, long-term (durable) left ventricular assist devices (LVADs) have emerged as a crucial lifeline for individuals battling advanced left ventricular failure. As the prevalence of heart failure continues to rise, so does the need for innovative approaches to diagnose and manage this debilitating condition. Multimodality imaging has proven to be a game-changer, enabling clinicians to comprehensively assess durable LVAD function and optimize patient outcomes. This editorial delves into the applications of multimodality imaging in the context of durable LVADs, highlighting its importance in the management of advanced heart failure.

## A holistic multimodality imaging approach to durable LVAD patients

Multimodality imaging techniques, such as echocardiography, computed tomography (CT), cardiac magnetic resonance imaging (MRI), and nuclear studies, offer distinctive advantages when it comes to assessing LVAD function. Echocardiography remains the primary modality for initial assessment due to its accessibility, real-time visualization, and ability to assess key parameters such as blood flow, device position, and the presence of complications like thrombosis, right heart failure, or tamponade etc. (1, 2). Moreover, cardiac MRI and CT play pivotal roles in providing additional valuable insights. A holistic multimodality imaging approach using echocardiography, CT, MRI and nuclear studies, plays a vital role in the assessment of durable left ventricular assist devices (LVADs) in advanced heart failure patients (1, 3).

## Echocardiography—the imaging gateway for LVAD assessment

Echocardiography remains a fundamental tool in LVAD assessment due to its real-time imaging capabilities, bedside availability, and ability to provide immediate insights into LVAD function, pump speed optimisation, inflow cannula/outflow graft velocities, and assessment of native heart structure and function. It aids in evaluating ventricular size, contractile function, valvular function, and can guide decision-making regarding the potential for myocardial recovery (3).

Doppler echocardiography assists in evaluating blood flow patterns within the LVAD, quantifying valvular dysfunction severity, and non-invasively measuring various hemodynamic parameters. It contributes to evaluating the effectiveness of medical management and the need for valve interventions (5).

Echocardiography is invaluable during LVAD implantation procedures, providing real-time guidance for surgeons regarding device positioning, cannula placement, ruling out intracardiac shunts or intracardiac thrombi. Moreover, echocardiography is vital in evaluating complications like pericardial effusion, cardiac tamponade and right ventricular failure (1). It is also vital for postoperative monitoring, identifying device malfunction, assessing ventricular recovery and detecting complications such as LVAD thrombosis or infection (1). LVAD suction events can develop due to RV failure, hypovolaemia, excessive LVAD pump speed or tamponade and all these aetiologies could be readily assessed at the bedside using echocardiography which is also essential during resuscitation and troubleshooting during cardiac arrest (3, 6). Furthermore, point of care ultrasound assessment of congestion could enhance standard echocardiographic assessment as lung ultrasound can provide useful insights in case of elevated extravascular lung water. Moreover, ultrasound markers of systemic venous congestion could provide valuable insights on right ventricular preload.

Serial echocardiographic examinations are crucial for long-term follow-up and surveillance of LVAD patients, enabling the monitoring of LVAD function, assessment of ventricular size/function, and detection of complications. Multimodality integrated use of the aforementioned imaging modalities provides a comprehensive assessment of LVAD patients (3).

## Cardiac MRI—a window to detailed evaluation

Cardiac MRI has emerged as a potentially useful tool for evaluating LVAD patients before implantation and after explantation. It provides high-resolution images, allowing for precise assessment of myocardial function, and tissue characterization. By employing cine imaging and late Gadolinium enhancement, clinicians can evaluate for myocardial fibrosis or inflammation. Unfortunately, there are no MRI-compatible LVAD devices in use, therefore the role of MRI is limited to assessment before implantation and after explantation (5). MRI would be indeed highly useful for detection and evaluation of LVAD-related complications (particularly in patients with inflow, intra-pump and outflow pump thrombosis), but unfortunately the risks are too high.

## Computed tomography—a comprehensive visualization

Computed tomography complements echocardiography and cardiac MRI by offering detailed anatomical visualization. It aids in the assessment of vascular anatomy, cannula position, and potential complications like outflow graft obstruction or kinking. Furthermore, CT assists in planning for LVAD implantation or explantation procedures, providing a roadmap for surgeons and optimizing patient outcomes.

## Nuclear studies—towards a refined assessment

Nuclear scan assists in the diagnosis of infection in LVAD patients by assessing the uptake of the glucose-analoge and radiotracer 18-fluorodeoxyglucose (FDG). Several studies have demonstrated that FDG-PET/CT can accurately localize the site and extent of the late LVAD infection across the peripheral driveline and the involvement of the central portion of the pump. Moreover, it has a higher ability to predict outcomes of LVAD infection than CT scan (7).

## Considerations and future directions

While multimodality imaging proves to be immensely valuable, certain considerations must be kept in mind. Standardization of protocols and reporting guidelines are crucial to ensure consistency and facilitate meaningful comparisons across studies. Additionally, efforts should be made to reduce radiation exposure associated with cardiac CT, especially in patients who may require multiple scans during their LVAD journey.

Looking ahead, the integration of artificial intelligence (AI) and machine learning algorithms holds immense promise in refining LVAD assessment. These technologies can analyse large datasets, identify subtle changes in LVAD function, and predict adverse events. Additionally, advancements in imaging hardware and software will continue to enhance image quality, reducing acquisition times and patient discomfort.

## Conclusion

Multimodality imaging has revolutionised the assessment of long-term (durable) LVADs, empowering clinicians in their mission to optimize the care of patients with advanced heart failure. By combining the strengths of echocardiography, cardiac MRI, CT and nuclear studies, clinicians can comprehensively evaluate LVAD function, detect complications, and tailor treatment strategies accordingly. With ongoing advancements in imaging technology and the integration of AI, the future of multimodality imaging looks brighter than ever, promising improved outcomes and enhanced quality of life for those battling advanced heart failure.
